# Using Statistical Methods to Comparatively Analyze the Visual Characteristics of Flattened Bamboo Boards in Different Bamboo Culms

**DOI:** 10.3390/polym14204327

**Published:** 2022-10-14

**Authors:** Caiping Lian, Xuehua Wang, Hong Chen, Benhua Fei, Xiaoren Pang, Jianchang Lian, Zhihui Wu

**Affiliations:** 1 College of Furnishings and Industrial Design, Nanjing Forestry University, Nanjing 210037, China; 2 Jiangsu Co-Innovation Center of Efficient Processing and Utilization of Forest Resources, Nanjing 210037, China; 3 International Center for Bamboo and Rattan, Beijing 100102, China; 4 Zhejiang Shenghua Yunfeng Greeneo Co., Ltd., Deqing 313220, China; 5 Long Bamboo Technology Group Co., Ltd., Nanping 353000, China

**Keywords:** flattened bamboo boards, visual physical properties, visual psychological quantities, comparison analysis, correlation analysis, multiple linear regression analysis

## Abstract

Different flattened bamboo boards will produce different visual effects, which directly determine consumers’ preferences. However, their visual characteristics were unknown. To clarify the visual effects of flattened bamboo boards in different bamboo culms, the visual, physical, and psychological quantities were firstly studied using their quantitative color and glossiness measurements, combined with quantitative semantic differential and statistical methods. Key results revealed that the values of lightness L* and blue–yellow index b* from the base to the top of the bamboo culms tended to decrease gradually, while green–red index a* values exhibited an increasing trend, and glossiness *G_ZL_* (*G_ZT_*) showed no significant difference. The L* value of bamboo outer layer (30.18) was smaller than that of the outer (61.90) and the inner (68.68), which had an increasing trend from the outside to the inside of the bamboo culm, while the *G_ZL_* (*G_ZT_*) values corresponded to 6.07 (4.66), 4.51 (3.12), and 2.77 (2.55), showing an opposite trend. The a* and b* values present a rise-fall tendency. According to visual psychological assessment, the outer was reflected as an “artificial–decorative”, “smooth–warm”, and “comfort–sophisticated” feeling; the inner had an “artificial–practical”, “smooth–warm”, and “comfort–sophisticated” sense; the bamboo outer layer had an “nature–practical”, “rough–cold”, and “sick–primitive” sense. Furthermore, predictive models for visual psychological quantities were constructed. This work provides a theoretical data basis for furniture design and standard materials application of flattened bamboo boards.

## 1. Introduction

As a kind of green material for protecting the environment, bamboo has attracted increasing attention due to its fast speed of growing, high yield, and superior mechanical properties [1,2,3]. Bamboo culm is used for manufacturing natural fibers, bio-composites, bamboo green products, etc. [4], which are essential elements in the production of green furniture [5,6]. Boran et al. [7] proposed bamboo as an ideal raw material for the green furniture industry, which can adhere to the principles of green or eco-friendly products. At present, bamboo is widely used in furniture and interior decoration in the form of round bamboo, laminated bamboo lumber, bamboo scrimber, sliced (rotated) thin bamboo, and other units [8,9]. However, the disadvantages of these traditional bamboo products are the low utilization rate of bamboo biomass or relatively high content of required adhesives, and high manufacturing costs [10], seriously restricting the healthy development of the bamboo processing industry. Through the progress of bamboo flattening technology, flattened bamboo boards could overcome these disadvantages as a new type of bamboo-based panel and increase the added value of bamboo [10,11].

**Figure 1:**
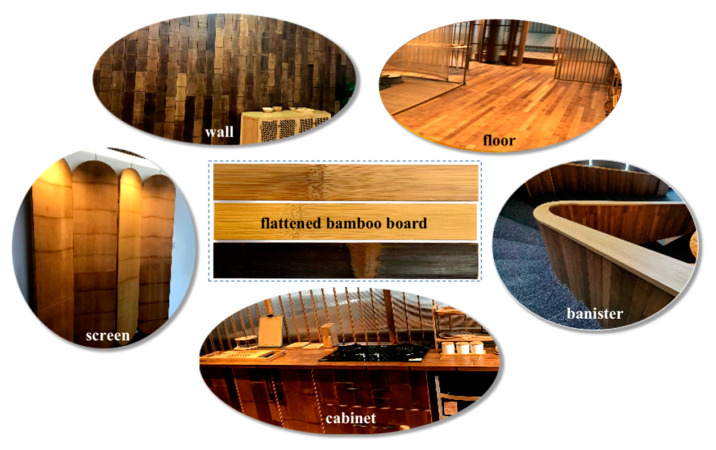
Applications of the flattened bamboo board in interior decoration and furniture.

Bamboo has long been regarded as a natural polymeric composite material, and its fibers are mainly used as reinforcement for natural fiber-reinforced polymeric composites [12]. Flattened bamboo boards are obtained from bamboo culms by softening and flattening at high temperature [13]. Hemicelluloses, lignin, and noncrystaline cellulose, which are polymeric amorphous constituents in the bamboo cell wall, exhibit viscoelastic behavior when exposed to this high temperature (glass transition temperature), allowing bamboo culms to flatten with pressure [14,15]. With the pyrolysis of a small part of these bio-polymers, the color of flattened bamboo boards is darker. Color is one of the important quality indexes of bamboo products that affect consumer preference and the added value of bamboo-based products [16,17]. In recent years, flattened bamboo boards have been gradually applied to interior decoration and furniture (Figure 1). Flattened bamboo board not only has high mechanical properties [15], but also maintains the natural texture of bamboo. In addition, as bamboo flattening technology can include or remove the bamboo outer layer [18], the flattened bamboo board has a variety of colors, providing a good decorative effect when used for furniture.

Environmental effects are highly important for green or ecological furniture products [7]. Bamboo materials, including flattened bamboo boards, have better ecological and carbon-friendly environmental characteristics, such as being renewable, biodegradable, absorbing carbon from the atmosphere, and producing less pollution [19,20]. As the advantages of flattened bamboo boards will inevitably lead to high application in the furniture and interior environment, their influence on the living environment must be determined. The utilization of woody materials in the living environment directly affects the comfort of human living and even individual health [21,22,23]. Therefore, it is particularly important to study the environmental characteristics of furniture materials. So far, research has predominantly focused on the flattening technology and mechanism and the properties of flattened bamboo board [11,14,15,24,25,26,27], while its environmental characteristics have not been addressed.

Environmental characteristics are the use of some objective physical quantities and subjective evaluation quantities to reflect the impact of the occupants’ feelings, psychology, and health, and to evaluate the habitability of the environmental space created by woody materials and their contribution to human comfort. They mainly include visual, tactile, and auditory characteristics [22] (Liu et al., 2008). People first pay attention to products’ visual characteristics when buying furniture products, so the visual properties of furniture materials directly affect the perception and satisfaction of consumers to furniture products. That is say, consumers’ visual perceptions of the surface characteristics of furniture materials significantly influence their overall evaluation of furniture [28,29]. The color and texture of the visual characteristics can produce enough visual stimulation to cause human psychological and physiological responses [30]. Additionally, because bamboo is a kind of gradient material, the properties of bamboo culms in different parts vary greatly, as bamboo culms gradually become denser from the base to the top [8,31] Especially from the outer to the inner walls, the fraction of the fibers and vascular bundles decrease gradually, and that of the ground tissue is reversed [32,33]. There is also a gradual decline of cellulose and liginin content from the outside to the inside of bamboo culms [34]. These factors result in certain differences in the color, gloss, and texture of the flattened bamboo boards. As variations affect the overall quality of bamboo furniture products and their visual effects, it is necessary to study the visual, physical, and psychological quantities of flattened bamboo boards in different parts of bamboo culms.

In this study, we characterized the quantitative color and glossiness measurements of flattened bamboo board surfaces in different parts, including the different internodes and radial bamboo walls of bamboo culms, using a colorimeter and a gloss meter. We also investigated and analyzed the subjective psychological quantities of the flattened bamboo boards by the semantic differential method (SD method). In addition, we analyzed the correlations between physical and psychological quantity. The aim of this work is to assist the high-value and efficient utilization of flattened bamboo boards in the application of the living environment, including furniture and interior decoration. We seek to provide scientific, environmental theory support for the design, manufacturing, and standard specifications of bamboo furniture products in the future to improve their utilization level and competitiveness.

## 2. Materials and Methods

### 2.1. Samples Preparation

**Figure 2:**
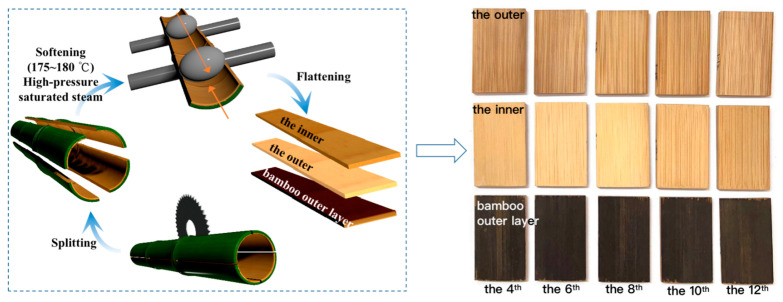
Schematic image of the sample preparation process and flattened bamboo board samples.

Six natural growing, three-year-old moso bamboos (*Phyllostachys edulis*) were collected from Nanping City, Fujian Province, China. Three were used to prepare the flattened bamboo boards with bamboo outer layers (keeping the bamboo green), and the other three were used to prepare the flattened bamboo boards without 1 mm thickness (removing bamboo green and yellow). The bamboo samples were split and cut into strips with a size of 2000 mm × 40 mm × 5 mm (long × wide × thick) from the 4th to the 12th internode. The flattened bamboo board samples were prepared by Fujian Longzhu Group Co., Ltd., by combining the flattening bamboo process with non-notched flattening technology. A total of 15 groups of knotless samples were produced, with 6 samples per group and 90 samples in total (Figure 2). The size of the samples was 60 mm × 40 mm × 5 mm (long × wide × thick). The samples were then air-dried to a moisture content of 8%.

### 2.2. Colorimetric Analyses

The color test was performed according to the color space CIE L*a*b* [35,36,37]. The color evolution of the 15 groups of samples was monitored using a color meter (X-Rite PANTONE series spectrophotometer, RM200, America) with a D65 standard light source. The observation angle was set at 10° and the optical aperture was fixed at 8.0 mm. Six measurements were performed on the smoothest surface of each sample. The average value of each group of the six flattened bamboo board samples was taken as the color determination value for this group of samples. The CIE L*a*b* 1976 system consists of three parameters L* (lightness), a* (green–red coordinate), and b* (blue–yellow coordinate) to describe the color of a the material surface. The hue-angle Ag* and color saturation C* are determined as follows:

(1) $$Ag*=\mathrm{arc}\tan(b^{*}/a^{*})180/\pi ;\;C*=\sqrt{a^{*2}+b^{*2}}$$

### 2.3. Glossiness Measurements

The surface glossiness of the flattened bamboo board samples was determined using a gloss meter (HG268, Shenzhen 3nh Technology Co., Ltd., Shenzhen, China) according to the ISO (2014) [38]. The chosen geometry was an incidence angle of 60°, and the results were based on a specular gloss value of 100 (this relates to the perfect condition under identical illuminating and viewing conditions of a highly polished plane) [39]. The glossiness value was measured at three points on each sample surface, and that of duplicate samples was averaged as the final value for each group of samples. The glossiness included *G_ZL_* and *G_ZT_* parameters. The former indicated that the light source was parallel to the texture direction of the material surface, and the later indicated that the light source was perpendicular to the texture direction of the material surface.

### 2.4. Visual Psychological Quantities Evaluation

The visual psychological quantity measurement was based on the approach adopted by [40]. The 50 participants in this experiment ranged in age from 18 to 26 years old and included 25 males and 25 females, all of whom were furniture design and engineering majors at Nanjing Forestry University.

The first step in the evaluation process was to collect and determine the semantic adjectives. The subjects first selected 30 pairs of semantic bipolar adjectives conforming to the visual Kansei images of the flattened bamboo boards from 45 pairs of meaningful semantic bipolar adjectives, collected by professionals according to their subjective consciousness (Table 1). They then classified the 30 pairs of bipolar adjectives according to sensory, emotional, and evaluative dimensions. Finally, according to the probability of adjectives selection, two pairs of representative adjectives were determined in each dimension for the visual stimulus questionnaires (Table 2).

Visual stimulus was performed by the SD method. This technique is useful for quantifying individual impressions and measures impressions of objects by using multiple pairs of semantic adjectives with antithetical meanings [41,42]. Based on the SD method, 15 samples were combined with the selected bipolar adjectives to form an evaluation scale with a 7 points scale (+3 to −3) that had an evaluation grade difference of 1 (Table 3). The experiment was conducted in a well-lit room, and the questionnaires, provided by Questionnaire Star, were presented on a phone screen. Subjects sat quietly, viewing the actual sample surface, while responding by clicking buttons on the screen corresponding to their answers.

### 2.5. Statistical Analyses

The color and glossiness data and SD data of the flattened bamboo boards measured in this experiment were processed by IBM SPSS 26 Statistics software. Correlation and multiple regression analysis between the visual physical quantities and psychological evaluation were also performed.

## 3. Results and Discussion

### 3.1. Visual Physical Quantities

**Figure 3:**
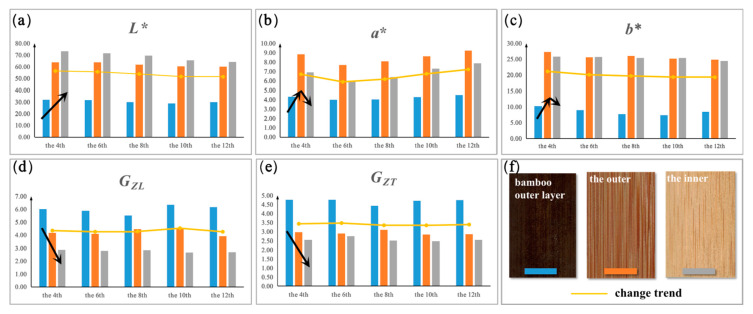
The changing treed and difference of visual physical quantities in different bamboo culm parts. (**a**–**e**) corresponding to variation trends of L*, a*, b*, *G_ZL_* and *G_ZT_* parameters, (**f**) the macro images of three types of flattened bamboo boards. Black arrow refers to the variation trend of each visual physical quantity among three types of boards; the orange dotted broken line indicates the change trend of each visual physical quantity at different internodes.

The quantified values of color and glossiness of flattened bamboo boards in different bamboo culm parts are shown in Table 4, while their changing trends are displayed visually in Figure 3.

The material color is the most important natural attribute that reflects the visual characteristics of an object’s surface and prompts human psychological feelings. The lightness, hue, and saturation of the material are the three basic characteristics of the color, and the latter two are determined by a* and b*. Therefore, these three parameters are usually used to analyze the color of a material surface [22]. Figure 3 (orange dotted broken line) shows that the values of L* (Figure 3a) and b* (Figure 3c) from the base to the top of bamboo culms tended to decrease gradually, while the a* values (Figure 3b) exhibited an increasing trend. It is also notable that from the base to the top, the overall results of color values of flattened bamboo board surfaces did not change significantly. The chromophore and auxochrome of chemical composition are known to affect the color of biomass material [43]. Jiang [32] found that there was little variability in chemical composition among the bamboo internodes. Therefore, when using flattened bamboo boards, we can ignore the influence of color variation caused by the height factor from sections 4–12.

The color difference from the outside to the inside of bamboo culms in the flattened bamboo boards was large (Figure 3), and could be recognized with the naked eye. The color of the bamboo outer layer was dark brown, that of the outer surface was reddish brown, and that of the inner surface was bright yellow. As yellow is the brightest color in the spectrum [44], the lightness of the bamboo outer layer and the outer was smaller than that of the inner. This is consistent with the measured color parameter values (Table 4). According to the black arrow in Figure 3, the L* values showed an increasing trend from the outside to the inside of the bamboo culm, and the a* and b* values have a rise-fall tendency. The color related chemical components in bamboo mainly arise from lignin and some extracts with unsaturated structures [35,45]. The content of lignin decreased gradually from the outside to the inside of bamboo culms [46]. With the softening and flattening treatment of bamboo at 180°, the lignin pyrolysis leads to the deepening of the color of the bamboo surface, which may be the main reason for the gradual enlargement of L* values from the bamboo outer layer to the inner. The hue Ag* values of all sample surfaces varied from 60~80, which indicated the flattened bamboo boards were warm-toned. In addition, the red color was mainly determined by the fiber sheath, as shown in the outer sample image in Figure 3. As the bamboo outer layer and the inner are mainly composed of parenchymal cells [31], the a* value of the outer was the largest.

Regarding glossiness, from the base to the top of bamboo culms, there was no significant difference in the flattened bamboo boards (Figure 3d,e). However, the glossiness of the bamboo outer layer was the highest (*G_ZL_*, 5.98%; *G_ZT_*, 4.68%), followed by the outer surface (*G_ZL_*, 4.23%; *G_ZT_*, 2.92%), and the lowest was the inner surface (*G_ZL_*, 2.76%; *G_ZT_*, 2.57%) (Table 4). In reality, the surface glossiness of bamboo is mainly related to its microstructure, thylose, and inclusions [22,35]. The outermost layer of the bamboo wall is the epidermis, which contains embolic and siliceous cells with high silica. From the outer to the inner, the cell structure changes according to a gradient, including a gradual reduction of vascular bundle density and the number of long cells and large cells (such as vessel elements). The silicon content of the bamboo wall also decreased gradually [31]. Thus, the scattering degree of reflected light on the cells slowly increases from the outer to the inner part of the bamboo culm. Additionally, it can be seen that the *G_ZL_* value of glossiness of all flattened bamboo samples parallel to the texture direction was greater than the *G_ZT_* value perpendicular to the texture direction (Figure 3d,e). This is because the cell long axis direction is the same as the bamboo texture, and the cell canal diameter is much smaller than the cell length. Therefore, when the light is parallel to the texture, it is less likely to be blocked by the inner wall of the cell than when it is incidentally perpendicular to the texture, and will improve the mirror reflectively of the bamboo in this direction [47].

### 3.2. Correlation Analysis of Visual Physical Quantities

As there is no linear relationship both the color parameters a* and b* among the outer, inner, or bamboo outer layer, the correlation analysis of the visual quantity parameters of the three types of boards was carried out, respectively. Table 5 shows the correlation between variables through the correlation matrix (correlation coefficient r). When 0 ≤ |r| ≤ 0.3, there is weak correlation; when 0.3 < |r| ≤ 0.5, there is low correlation; when 0.5 < |r| ≤ 0.8, there was a significant correlation; when 0.8 < |r| < 1, it is highly correlated [48]. The correlation analysis results between visual physical quantity parameters indicated that there are obvious correlations between multiple pairs of physical quantity parameters (Table 5). For instance, L* had a negative correlation with a*, and a significant positive correlation with b*, Ag*, and C* (0.5 < |r| < 1). The Ag* was significantly correlated with a* and b*. Although the C* was mathematically related to the a* and b*, C* was only highly positively related to b* (0.8 < |r| < 1), as the color of the bamboo was still yellow (b* > 0), and the values of b* in the flattened bamboo boards surface was greater than a*. Additionally, L* and the glossiness parameters (*G_ZL_*, *G_ZT_*) also had a low correlation (0 < |r| < 0.5). In conclusion, there is a certain degree of correlation between the visual physical quantity parameters, even at the level of 0.05. Therefore, the objective of simplifying the parameters can be achieved by retaining some of them.

The seven visual physical quantity parameters mainly reflect the brightness and color of the flattened bamboo board surface. Through the principal component analysis (Table 6) combined with correlation analysis (Table 5), the physical quantities parameters can be simplified as L*, b*, a* and *G_ZL_*. Since the values of Ag* and C* were calculated based on the values of a* and b*, and there is a good correlation between them (Table 5), the Ag* and C* parameters can be removed. Due to better factor-loading in principal component 1 (PC 1) and PC 2 (Table 6), b* and a* (with poor correlation with other parameters) were selected as the color principal component representatives. In terms of lightness, L* and the two glossiness parameters (*G_ZL_* and *G_ZT_)* express similar physical meanings, but L* was not strongly correlated with *G_ZL_* and *G_ZT_*. Meanwhile, the principal component analysis in Table 6 showed that L*, *G_ZL_*, and *G_ZT_* have higher factor loads in PC 1 and PC 2 of the three types of boards, respectively. As the length direction of flattened bamboo boards is generally parallel to the texture direction when it is actually used, the lightness L* and *G_ZL_* were retained as the representatives of the lightness principal components. To summarize, L*, b*, a*, and *G_ZL_* were taken as the comprehensive physical quantity parameters for the visual evaluation of the flattened bamboo board surface.

### 3.3. Psychological Quantities Expression

The American social psychologist Stanley Schachter proposed that the generation of emotion is determined by the environment, human psychology, and human physiology. The stimulation of the surrounding environment often causes variations in the physiology and psychology of an organism [23]. The different responses to the environmental stimuli in the human body are mainly expressed through the physiological aspects of vision, touch, audition, etc. [22]. Hence, a visual psychological evaluation is highly significant in environmental stimulus feedback.

**Figure 4:**
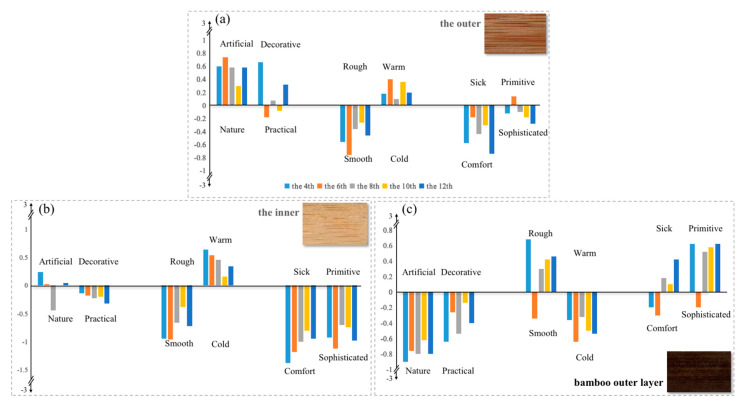
Distribution of visual psychological quantities in different parts of the bamboo culm. (**a**) The distribution in the outer surface. (**b**) The distribution in the inner surface. (**c**) The distribution in the bamboo outer layer surface.

The results of the psychological investigation (Figure 4) revealed that different internodes of flattened bamboo boards provided all subjects with a similar sense, which indicated that the visual characteristics of different internodes had no significant difference. This result was consistent with the previous measurement results on physical quantities. Among the three types of flattened bamboo boards, on the evaluative dimension (Figure 4a), the outer was reflected an “artificial–decorative” sense, while the bamboo outer layer surface had an “nature–practical” sense. Meanwhile, the inner surface provided an “artificial–practical” feeling. This was largely due to the variations in microstructure in the radial direction of the bamboo culms [32]. The bamboo outer layer and the inner were mainly composed of parenchyma cells, resulting in a single texture and color, which gave the samples a practical sense. Relatively speaking, the outer cell types, including a large number of vascular bundles and ground tissues [31], are diverse and could easily produce a red–yellow color and rich textures (Figure 3). Therefore, the outer of flattened bamboo board were more suitable for occasions requiring a strong decorative aspect. Regarding the sensory dimension (Figure 4b), both the outer and the inner surfaces provided a “smooth–warm” sense, which was related to the lightness L* and b*. Lightness is positively related to glossiness, and yellow gives people a warm feeling [49]. The bamboo outer layer gave a “rough–cold” feeling as its L* value was the lowest, which could easily impart a sense of roughness, and its b* and a* color was blue–green, which could give a cold feeling. For the emotional dimension (Figure 4c), most subjects felt that the outer and the inner were more comfortable and sophisticated. A few felt that the outer appeared a little primitive, while the bamboo outer layer gave most subjects a primitive and uncomfortable feeling. This investigation result suggested that the closer to the outside of bamboo culms, the stronger the sense of “primitive–sick”. To summarize, the outer and the inner of the flattened bamboo boards were generally suitable for bedrooms that needed to be warm, comfortable, and slightly decorative, while the bamboo outer layer of the flattened bamboo boards was more suitable for occasions that required a cold-primitive feeling.

Principal component analysis was performed on the visual psychological quantities of the six pairs of semantic adjectives used, with the goal of summarizing similar evaluation patterns among these flattened bamboo boards. Table 7 presents a summary of the items and loadings obtained by principal component analysis in each type of flattened bamboo boards. The three Kaiser–Meyer–Olkin (KMO) values were greater than 0.6, and the data passed Bartlett’s sphericity test (*p* < 0.05), indicating that the research data were suitable for principal component analysis. After considering the scree plot and cumulative contribution ratio after extraction (the outer: 82%; the inner: 86%; bamboo outer layer: 87%), four pairs of semantic adjectives (including practical–decorative, cold–warm, comfort–sick and sophisticated–primitive) were selected as principal components. However, as both “nature–artificial” and “smooth–rough” had high factor loadings, they were retained as the evaluation parameters of the visual psychological quantity of bamboo. Interestingly, although the factor loadings of the six pairs of semantic adjectives were different among the three flattened bamboo boards, their PC order was approximately the same. This also suggests that six affective properties adjectives for visually evaluating the flattened bamboo board surface are necessary. This result is consistent with the affective properties selected by [40] in the evaluation of bamboo filament.

### 3.4. Correlation and Multiple Regression Analysis of Between Comprehensive Physical and Psychological Quantities

According to the above statistical analysis of the visual physical and psychological quantities of the outer, the inner, and bamboo outer layer surfaces of flattened bamboo boards, the regular presented by the three types of boards was generally consistent. Therefore, the correlation analysis between four comprehensive physical quantities and six affective properties of the three types of boards were performed in a unified way, and the results were shown in Table 8.

As shown in Table 8, most physical and psychological quantities were significantly or highly correlated, while some were not. For example, the L* and *G_ZL_* values showed a significant correlation with the other five pairs of psychological quantities, except for “practical–decorative”. In particular, “smooth–rough” and “cold–warm” were highly correlated with the four comprehensive physical quantities. The a* value was significantly correlated with “nature–artificial”, “practical–decorative”, “smooth–rough” and “cold–warm”. The b* value had a significant correlation with all psychological quantities. Flattened bamboo boards with higher L*, b*, and a* gave a strong sense of decorative, smooth, warm, comfort, sophisticated, and artificial feeling. An improvement of the glossiness *G_ZL_* of the flattened bamboo board surface, such as the bamboo outer layer, will create a sense of nature, rough, cold, primitive, and even uncomfortable feelings. While this result was similar to that of the psychological quantities evaluation in Figure 4, the result of the bamboo outer layer was opposite to that of the physical quantity analysis. Yu et al. [23] proposed that the glossiness *G_ZL_* and lightness L* of wood plants are significantly positively correlated, whereby the glossiness would increase with the increase of lightness, and that the corresponding smoothness is also strong. However, for the bamboo outer layer, although the glossiness is the highest among all three types of boards, due to its extremely low brightness, it gave people a sense of darkness, which created a rough feeling that obscured the smoothness of the high glossiness. Therefore, the opposite phenomenon appears between the physical and psychological quantities in the bamboo outer layer.

Multiple linear regression analysis (MLR) was performed using the ridge regression method and stepwise method to further investigate which physical properties were related to which affective properties. The regression analysis results are shown in Table 9. All regression models passed the F-test (*p* < 0.05), and the variance inflation factor (VIF) for a “practical–decorative” independent variable was less than 1.4, and confirmed as unproblematic. Table 9 shows that the “nature–artificial” item was positively affected by L*, b* and a* (*p* < 0.05). The “practical–decorative” item only had a positive linear correlation with the a*. The “smooth–rough”, “comfort–sick” and “sophisticated–primitive” items were negatively affected by L* and b*, and positively influenced by the *G_ZL_*. Moreover, the “cold–warm” item was positively significant related with L* and b* and negatively highly related with *G_ZL_*. Thus, the mathematical correlation model of visual comprehensive physical quantities and psychological quantities could be constructed by the corresponding regression coefficient (Table 10). These result models could then be employed to predict the visual environmental characteristics of flattened bamboo boards under the condition that the visual physical quantity parameters of the flattened bamboo board surface have been measured and could then be used to evaluate the visual effect of a product surface [23].

## 4. Conclusions

By investigating the visual, physical, and physiological quantities and responses of flattened bamboo boards surfaces in different bamboo culms, the findings of this study provided scientific guidance for the use of different flattened bamboo boards, and reference models to evaluate the effects that flattened bamboo boards’ product surface characteristics exert on consumers’ visual cognition. The following conclusions were obtained:

The comparative analysis illustrated that the color parameters decreased or increased regularly in the longitudinal direction of the bamboo culms, but the change was small. However, the color and glossiness parameters in the radial direction of the bamboo culms were significantly different. In conclusion, the color range of all flattened bamboo boards surfaces was mainly yellow–brown with a warm-toned hue. Additionally, four comprehensive physical parameters, including L*, a*, b*, and *G_ZL_*, were obtained by correlation and principal component analysis to evaluate the visual effect.

For visual physiological quantities, the visual characteristics of different internodes had no significant difference, but that of different parts in the radial direction of bamboo culms had a significant difference. The outer and inner surfaces created smooth–warm and comfort–sophisticated feelings, and were suitable for use in bedrooms, while the bamboo outer layer produced a rough–cold and sick–primitive feeling. In addition, the outer surface had a decorative capacity. Principal component analysis suggested that “nature–artificial”, “practical–decorative”, “smooth–rough”, “cold–warm”, “comfort–sick”, and “sophisticated–primitive” were necessary parameters to evaluate the visual characteristics of flattened bamboo boards surfaces.

The L*, a*, and b* were positively correlated with “nature–artificial”, “practical–decorative”, and “cold–warm”, and negatively correlated with “smooth–rough”, “comfort–sick”, and “sophisticated–primitive”. The correlation between the GZL and six pairs of semantic bipolar adjectives was the opposite. Therefore, the linear regression models between psychological quantity and comprehensive physical quantity were constructed by multiple linear regression.

Due to the subjectivity of the psychological quantity evaluation and singleness of samples, future works will include the following aspects: (1) the study will be combined with some characterization methods, such as eye tracking and electroencephalogram (ERP), to objectively express the visual characteristics of flattened bamboo boards. (2) Future studies will invite participants with a broader range of backgrounds, rather than only current university students as study subjects. (3) Due to the diversity of furniture materials, it is necessary to carry out a comparative study on the visual characteristics of flattened bamboo boards and other bamboo-based panels, including glued-laminated bamboo and bamboo scrimber.

## Figures and Tables

**Table 1:** Bipolar adjectives of semantic differentials selected for the visual Kansei images of flattened bamboo board samples.

NO.	Semantic Bipolar Adjectives	NO.	Semantic Bipolar Adjectives	NO.	Semantic Bipolar Adjectives
1	Nature–artificial	11	Whole–scattered	21	Sophisticated–primitive
2	Light–heavy	12	Slender–heavy	22	Continuous–discontinuous
3	Cold–warm	13	Dense–sparse	23	Comfort–sick
4	Easy–complicated	14	Soft–tough	24	Straight–bent
5	Elegant–plain	15	Streamlined–geometrical	25	Line–curve
6	Fluent–stagnant	16	Smooth–rough	26	complete–incomplete
7	Abundant–dull	17	fine–rugged	27	Common–rare
8	Harmonious–disorder	18	Soft–hard	28	Handsome–restrained
9	Clean–mussy	19	Fashion–classical	29	Clear–dull
10	Balanced–unbalanced	20	Tuneful–clutter	30	Practical–decorative

**Table 2:** Results of typical semantic bipolar adjectives in three dimensions.

Evaluative Dimension	Sensory Dimension	Emotional Dimension
Nature–artificial	Smooth–rough	Comfort–sick
Practical–decorative	Cold–warm	Sophisticated–primitive

**Table 3:** Semantic differential rating scale.

Degrees	Very Much	Considerably	Somewhat	Neither	Somewhat	Considerably	Very Much
Scales	−3	−2	−1	0	1	2	3

**Table 4:** The results of color and glossiness of flattened bamboo board surfaces.

	Samples	L*	a*	b*	Ag*	C*	*G_ZL_* (%)	*G_ZT_* (%)
The outer	the 4th	63.67	8.82	27.23	71.92	28.69	4.17	2.95
	the 6th	63.70	7.66	25.52	72.99	26.70	4.09	2.89
	the 8th	61.74	8.06	25.97	72.55	27.29	4.45	3.09
	the 10th	60.28	8.62	25.17	70.73	26.68	4.54	2.84
	the 12th	60.10	9.22	24.79	69.19	26.56	3.89	2.85
The inner	the 4th	73.21	6.91	25.82	74.69	26.82	2.85	2.56
	the 6th	71.31	5.95	25.64	76.55	26.39	2.78	2.75
	the 8th	69.50	6.39	25.34	75.55	26.18	2.83	2.52
	the 10th	65.49	7.28	25.40	73.04	26.57	2.67	2.48
	the 12th	63.96	7.87	24.45	71.44	25.85	2.68	2.56
Bamboo outer layer	the 4th	31.82	4.23	10.54	64.40	11.53	5.99	4.77
	the 6th	31.22	3.96	9.02	63.83	10.05	5.87	4.76
	the 8th	29.91	4.02	8.13	61.87	9.21	5.52	4.43
	the 10th	29.15	4.48	8.24	60.54	9.53	6.35	4.70
	the 12th	29.85	4.50	8.47	60.50	9.66	6.16	4.75

**Table 5:** Correlation analysis of seven visual physical quantities.

Samples	Parameter	Average	SD	Correlation Coefficient r (Pearson)						
				L*	a*	b*	Ag*	C*	*G_ZL_*	*G_ZT_*
The outer	L*	61.90	3.25	1						
	a*	8.48	1.17	−0.546	1					
	b*	25.74	1.63	0.733	−0.083	1				
	Ag*	71.48	2.86	0.806	−0.878 *	0.549	1			
	C*	27.19	1.50	0.597	0.151	0.972 **	0.34	1		
	*G* *_ZL_*	4.51	0.82	−0.152	−0.313	0.138	0.333	0.06	1	
	*G* *_ZT_*	3.12	0.62	0.317	−0.393	0.525	0.589	0.445	0.356	1
The inner	L*	68.68	6.82	1						
	a*	6.89	1.37	−0.743	1					
	b*	25.35	2.67	0.848	−0.709	1				
	Ag*	74.25	4.34	0.829	−0.989 **	0.775	1			
	C*	26.39	2.35	0.651	−0.312	0.890 *	0.404	1		
	*G* *_ZL_*	2.77	0.24	0.910 *	−0.661	0.653	0.752	0.414	1	
	*G* *_ZT_*	2.55	0.29	0.446	−0.608	0.241	0.57	−0.026	0.232	1
Bamboo outer layer	L*	30.18	3.85	1						
	a*	4.16	1.07	−0.247	1					
	b*	8.82	3.89	0.910 *	0.153	1				
	Ag*	61.98	7.53	0.786	−0.179	0.688	1			
	C*	9.91	3.83	0.918 *	0.138	0.982 **	0.798	1		
	*G* *_ZL_*	6.07	0.83	−0.336	0.703	−0.057	−0.65	−0.154	1	
	*G* *_ZT_*	4.66	0.31	0.399	0.484	0.568	−0.007	0.509	0.707	1

*. Significantly correlated at the 0.05 level; **. Significantly correlated at the 0.01 level.

**Table 6:** Principal component analysis of seven visual physical quantities. - indicates no value.

	The Outer			The Inner			Bamboo Outer Layer			Comprehensive Physical Quantities
	PC 1	PC 2	PC 3	PC 1	PC 2	PC 3	PC 1	PC 2	PC 3	
L*	0.811	−0.128	−0.221	0.821	−0.295	−0.398	0.921	0.110	0.276	L*
*G_ZL_*	0.072	0.872	−0.061	0.742	−0.259	0.480	−0.736	0.363	0.366	*G_ZL_*
*G_ZT_*	−0.014	0.841	−0.295	0.688	−0.457	0.425	−0.616	0.506	0.505	-
b*	0.849	0.116	0.502	0.85	0.518	0.082	0.962	0.143	0.13	b*
Ag*	0.938	−0.054	−0.283	0.972	0.055	−0.189	0.866	−0.207	0.386	-
C*	0.713	0.156	0.678	0.782	0.604	0.133	0.957	0.214	0.078	-
a*	−0.727	0.146	0.640	−0.871	0.249	0.356	0.418	0.751	−0.498	a*
Eigenvalues	3.302	1.545	1.341	4.734	1.062	0.756	4.536	1.072	0.885	6.411
% of Variance	47.167	22.075	19.162	67.623	15.176	10.803	64.793	15.309	12.637	91.58%
KMO	0.551			0.703			0.628			-
*p* value	0.000			0.000			0.000			-

**Table 7:** Principal component analysis of psychological quantities.

	The Outer				The Inner				Bamboo Outer Layer			
	PC 1	PC 2	PC 3	PC4	PC 1	PC 2	PC 3	PC4	PC 1	PC 2	PC 3	PC4
Nature–artificial	0.433	0.654	−0.028	0.52	−0.077	0.783	0.069	0.601	0.15	0.749	−0.348	0.527
Practical–decorative	0.231	0.790	0.341	−0.131	0.005	0.689	0.582	−0.425	0.084	0.785	0.219	−0.56
Smooth–rough	0.587	−0.319	0.662	−0.184	0.785	−0.153	0.306	0.084	0.824	−0.062	−0.244	−0.217
Cold–warm	0.611	0.308	−0.438	−0.549	0.283	0.619	−0.634	−0.297	0.426	0.069	0.835	0.293
Comfort–sick	0.694	−0.373	−0.199	0.269	0.833	0.164	−0.083	0.097	0.807	−0.044	−0.034	0.177
Sophisticated–primitive	0.715	−0.289	−0.07	0.086	0.846	−0.16	0.012	−0.016	0.883	−0.138	−0.106	−0.136
Eigenvalues	1.951	1.471	0.792	0.703	2.112	1.548	0.845	0.646	2.321	1.208	0.939	0.774
% of Variance	32.51	24.509	13.197	11.714	35.208	25.807	14.083	10.774	38.684	20.132	15.645	12.903
KMO	0.622				0.653				0.668			
*p* value	0.000				0.000				0.000			

**Table 8:** Correlation analysis between physical and psychological quantities.

	Nature–Artificial	Practical–Decorative	Smooth–Rough	Cold–Warm	Comfort–Sick	Sophisticated–Primitive
L*	0.779 **	0.478	−0.890 **	0.961 **	−0.841 **	−0.826 **
a*	0.922 **	0.727 **	−0.624 *	0.726 **	−0.489	−0.474
b*	0.864 **	0.580 *	−0.836 **	0.920 **	−0.750 **	−0.734 **
*G_ZL_*	−0.573 *	−0.270	0.854 **	−0.897 **	0.891 **	0.914 **

*. Significantly correlated at the 0.05 level; **. Highly correlated at the 0.01 level.

**Table 9:** Ridge and stepwise regression analysis results between comprehensive physical and psychological quantities.

Dependent Variable	Independent Variable	B	*t*	*p*	R^2^	F
Nature–artificial	constant	−2.444	−5.547	0.000 **	0.878	F (4,10) = 17.976, *p* = 0.000
	L*	0.007	2.407	0.037 *		
	a*	0.167	4.356	0.001 **		
	b*	0.023	3.966	0.003 **		
	*G_ZL_*	0.095	1.772	0.107		
Practical–decorative (stepwise method)	constant	−0.956	−4.356	0.001 **	0.528	F (1,13) = 14.564, *p* = 0.002,VIF =1
	L*	-	-	-		
	a*	0.123	3.816	0.002 **		
	b*	-	-	-		
	*G_ZL_*	-	-	-		
Smooth–rough	constant	0.058	0.263	0.798	0.613	F (4,10) = 6.539, *p* = 0.007
	L*	−0.007	−5.132	0.000 **		
	a*	−0.021	−1.105	0.295		
	b*	−0.011	−4.35	0.001 **		
	*G_ZL_*	0.085	3.615	0.005 **		
Cold–warm	constant	−0.326	−2.425	0.036 *	0.845	F (4,10) = 13.680, *p* = 0.000
	L*	0.006	7.249	0.000 **		
	a*	0.025	2.241	0.049 *		
	b*	0.01	6.514	0.000 **		
	*G_ZL_*	−0.067	−4.722	0.001 **		
Comfort–sick	constant	−0.407	−1.756	0.110	0.691	F (4,10) = 5.581, *p* = 0.013
	L*	−0.006	−4.668	0.001 **		
	a*	−0.002	−0.091	0.930		
	b*	−0.009	−3.335	0.008 **		
	*G_ZL_*	0.106	4.323	0.002 **		
Sophisticated–primitive	constant	−0.418	−1.279	0.230	0.76	F (4,10) = 7.929, *p* = 0.004
	L*	−0.008	−4.437	0.001 **		
	a*	0.017	0.601	0.561		
	b*	−0.01	−2.685	0.023 *		
	*G_ZL_*	0.175	4.836	0.001 **		

*. Significantly correlated at the 0.05 level; **. Highly correlated at the 0.01 level.

**Table 10:** Multiple linear regression models for visual psychological quantity prediction.

Psychological Quantities	Regression Equation
Nature–artificial	−2.444 + 0.007L* + 0.167a* + 0.023*G_ZL_*
Practical–decorative	−0.956 + 0.123a*
Smooth–rough	−0.007L* − 0.011b* + 0.085*G_Z__L_*
Cold–warm	−0.326 + 0.006L* + +0.025a* + 0.01b* − 0.067*G_Z__L_*
Comfort-sick	−0.006L* − 0.009b* + 0.106*G_ZL_*
Sophisticated–primitive	−0.008L* − 0.01b* + 0.175*G_ZL_*

## Data Availability

The raw data presented in this study are available on request from the corresponding author.
